# Peritoneal Tuberculosis With Raised Cancer Antigen 125 (CA-125) in a Post-partum Woman: A Case Report

**DOI:** 10.7759/cureus.35757

**Published:** 2023-03-04

**Authors:** Mayank Mahajan, Manoj K Prasad, Vidyapati Vidyapati

**Affiliations:** 1 Medicine, Rajendra Institute of Medical Sciences, Ranchi, IND

**Keywords:** cancer antigen 125, malignancy, ca-125, post-partum, peritoneal tuberculosis, case report

## Abstract

Peritoneal tuberculosis is a common cause of ascites in areas endemic to *Mycobacterium tuberculosis*. The presentation of tuberculous ascites can mimic ovarian malignancy when it is associated with elevated cancer antigen 125 (CA-125) levels. We hereby discuss a case of a four months post-partum female patient who presented with gradual abdominal distension and was diagnosed with peritoneal tuberculosis after proper evaluation. She was started on anti-tubercular therapy and the treatment was successful. This case report highlights the importance of considering peritoneal tuberculosis as a differential diagnosis in cases of ascites with raised serum CA-125 levels in a *Mycobacterium tuberculosis *endemic region.

## Introduction

Tuberculosis (TB) is an infectious bacterial disease caused by the agent *Mycobacterium tuberculosis*. When it affects organs outside the lungs, it is known as extrapulmonary TB, and it accounts for around 15% of TB cases [1]. Out of these, around 11% of cases comprise abdominal TB, which may lead to ascites [2]. Tuberculous ascites is characterised by non-specific signs and symptoms like fever, nausea, vomiting, weight loss, abdominal pain, and abdominal distension. The diagnosis of tuberculous ascites is based on clinical features, radiology, and ascitic fluid examination. Ascitic fluid in TB is exudative [3], which is also found in cases of ascites due to malignancy.

Cancer antigen 125 (CA-125) is a tumour marker useful in the diagnosis of ovarian cancer [4,5]. CA-125 is a glycoprotein, which is expressed by the cells lining the uterine endometrium and the serum levels are raised in conditions like ovarian malignancy, endometriosis, and pelvic inflammatory disease [6,7]. CA-125 is also expressed by the cells lining the pleura, pericardium, and peritoneum and thus the serum levels may be elevated in cases of tuberculous peritonitis, intestinal malignancies, and in postoperative cases [8-12]. Due to similar clinical manifestations along with the ascitic fluid examination and high serum CA-125 levels, tuberculous ascites may often be confused with malignancy [13]. This is particularly a problem in female patients with ascites, where the diagnosis becomes challenging. Although in Western countries, malignancy and liver cirrhosis are the common causes of ascites, in tropical countries like India, TB is relatively more prevalent. There have been many reports of cases of tuberculous ascites mimicking ovarian cancer [14,15]. But there is no specified cut-off value for serum CA-125 level, which can differentiate between tuberculous ascites and ovarian malignancy. Timely diagnosis and treatment can decrease morbidity and mortality in such cases. Thus, we hereby present a case of a female patient with post-partum development of ascites and raised serum CA-125 levels, who was diagnosed with peritoneal TB.

## Case presentation

A 25-year-old female was admitted to our hospital with a complaint of gradual distension of the abdomen for four months associated with weight loss. The distension was painless and progressive and was not associated with fever, vomiting, or constipation. There was no history of any chronic illness, alcohol abuse, intake of any suspected drug/herbal agent, or similar illness in the family. The patient underwent a lower segment caesarean section (LSCS) procedure for the delivery of the baby four months back, and around the same time, the patient started noticing abnormal distension in her abdomen.

On examination, the vitals of the patient were normal (pulse rate: 96 beats per minute; blood pressure: 106/62 mmHg). The patient was afebrile, and she had normal auscultatory findings for the lungs and cardiovascular system. Abdominal examination revealed ascites with shifting dullness and fluid thrill. Laboratory investigations are shown in Table 1.

**Table 1:** Laboratory investigations Laboratory investigations of the patient after admission.

Parameters	Results	Reference range
Haemoglobin	10.1 g/dL	12.5-16.0 g/dL
Total leukocyte count	5.4 thousand/µL	4.0-11.0 thousand/µL
Platelet count	2.52 lakh/µL	1.5-4.5 lakh/µL
Erythrocyte sedimentation rate	14.0 mm/hour	0-14.0 mm/hour
Urea	18.6 mg/dL	15-40 mg/dL
Creatinine	0.7 mg/dL	0.6-1.4 mg/dL
Sodium	136 mEq/L	135-145 mEq/L
Potassium	3.8 mEq/L	3.5-4.5 mEq/L
Calcium	10.1 mg/dL	8.5-10.5 mg/dL
Random blood glucose	96 mg/dL	<140 mg/dL
Total bilirubin	0.8 mg/dL	0.2-1.2 mg/dL
Serum glutamic oxaloacetic transaminase (SGOT)	16 U/L	<45 U/L
Serum glutamic pyruvic transaminase (SGPT)	23 U/L	<40 U/L
Alkaline phosphatase (ALP)	188 U/L	80-290 U/L
Total protein	6.9 g/dL	6.0-8.0 g/dL
Albumin	4.1 g/dL	3.7-5.3 g/dL
Cancer antigen 125 (CA-125)	154.4 U/mL	<35.0 U/mL

**Figure 1:**
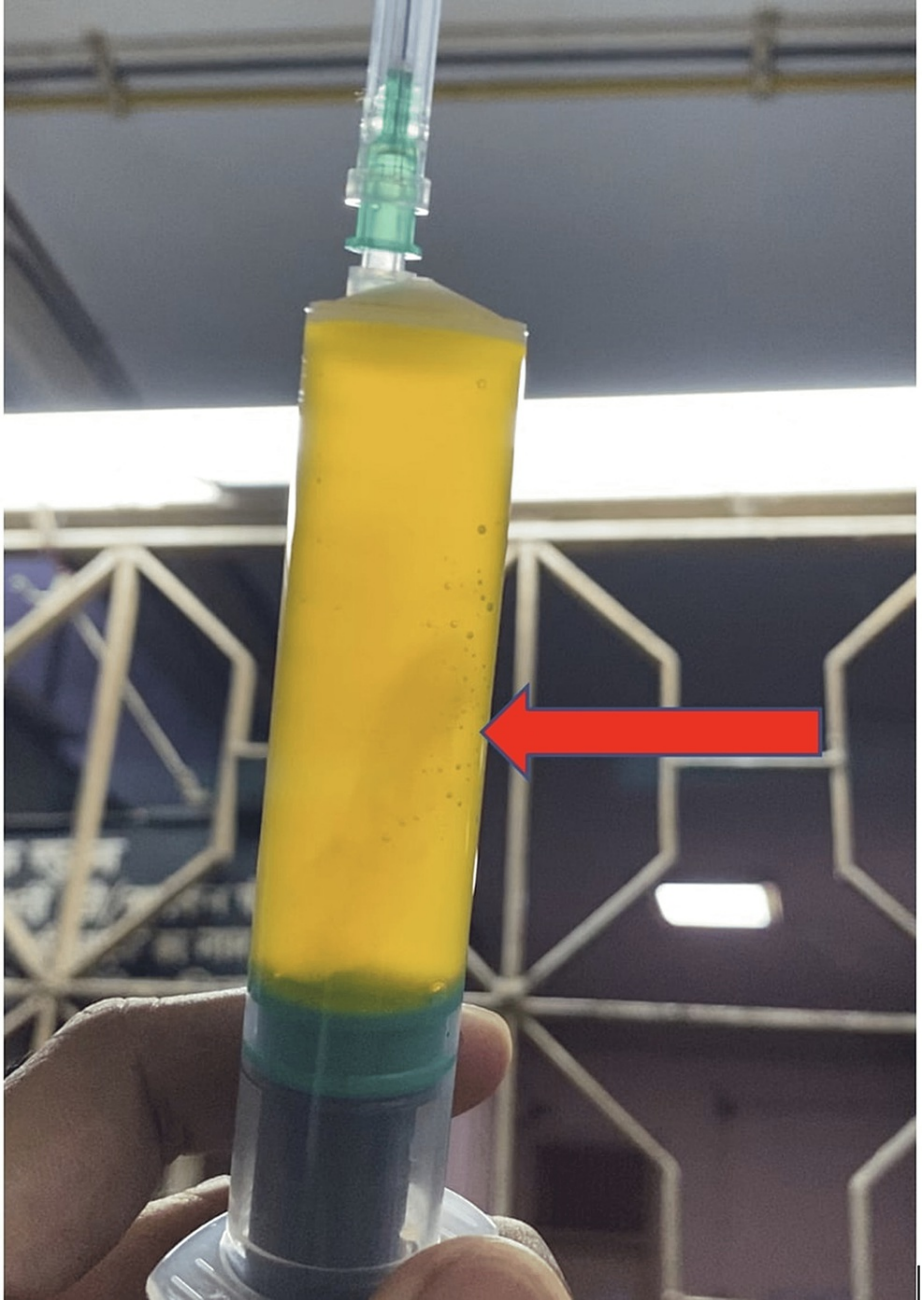
Ascitic fluid of the patient The straw-coloured ascitic fluid of the patient obtained after paracentesis, as shown by the red arrow.

Other investigations included a thyroid function test, which was normal, and viral markers for human immunodeficiency virus (HIV) and hepatitis A, B, C, and E, which were all negative. A routine examination of urine was also normal and did not show any proteinuria. Her chest X-ray was completely normal with no infiltrations or effusions. Two-dimensional echocardiography was also normal with normal ejection fraction. Ultrasonography of the abdomen and pelvis region reported gross ascites, and all organs, including the reproductive tract, were normal in structure, with no evidence of malignancy. We performed paracentesis on this patient and obtained a straw-coloured ascitic fluid, which was further sent for pathological examination (Figure 1).

The report showed a total white cell count of 581 cells/µL with 80% lymphocytes, 3.5 gm% of albumin, and the absence of malignant cells. According to these results, the serum ascites albumin gradient (SAAG) was 0.6, which showed that the fluid was exudative. Direct microscopic examination of ascitic fluid along with staining for acid-fast bacilli was done and found to be negative. Ascitic fluid culture also failed to demonstrate *Mycobacterium tuberculosis* bacteria. Ascitic fluid adenosine deaminase (ADA) level was elevated to 32 U/L (reference range: 0-30 U/L). Although malignancy was important for differential diagnosis due to raised CA-125 levels, other results strongly supported TB. We sent a repeat sample of peritoneal fluid for malignant cells examination and the report was negative. The patient was started on four-agent TB treatment (isoniazid, rifampicin, pyrazinamide, and ethambutol) and a short course of diuretics (furosemide and spironolactone). The patient came for regular follow-up over months and she had a good clinical response, with a resolution of ascites over time.

## Discussion

Peritoneal TB is a disease of concern, especially in developing countries. It presents with non-specific signs and symptoms like fever, weight loss, abdominal pain, and ascites. Laboratory investigations and radiological imaging are also non-specific. Ascitic fluid is exudative and may be associated with elevated serum CA-125 levels [12]. This makes it difficult to differentiate it from abdominal malignancy, especially ovarian carcinoma. Thus, clinical suspicion is very much essential for diagnosing peritoneal TB in a case of a female patient presenting with ascites and raised serum CA-125 levels.

In our case, there were no symptoms suggestive of TB, except ascites and weight loss. There was no history of cough or hemoptysis suggesting pulmonary TB. There was no history of contact with any TB patient, and the chest X-ray was also normal. There was a history of LSCS operation four months back after which the patient developed ascites. Ascitic fluid analysis revealed increased leucocytes with lymphocytic predominance, but staining for acid-fast bacilli was negative. Ascitic fluid ADA has emerged as a useful marker for peritoneal TB. Fortunately, in our case, the ADA level was elevated, which inclined our diagnosis toward TB. Extrapulmonary TB usually develops by the hematogenous or lymphatic spread of the TB bacteria. But sometimes, the infection may directly spread from an adjacent affected organ. This is particularly a problem in female patients where infection may spread to the peritoneum from abdominal lymph nodes or from the female reproductive system.

In our case, the patient underwent an operative procedure for LSCS four months back, which may have caused the seeding of TB bacteria from the reproductive tract to the peritoneum, leading to peritoneal TB and ascites. Factors affecting the immune system of the body like HIV, diabetes, and tobacco smoking further increase the risk of getting the infection. In this case, the patient was in a post-partum and post-operative state when the immunity was low and the body was weak. The patient was started on anti-tubercular therapy and she recovered successfully after the course of treatment.

## Conclusions

Peritoneal TB can mimic malignancy and cause a delay in diagnosis due to lack of suspicion. Timely diagnosis with proper treatment can help to prevent morbidity and mortality. In this case, the patient presented with ascites and raised serum CA-125 level creating a suspicion for malignancy like ovarian cancer. The ascitic fluid was exudative with raised ascitic fluid ADA level. There was no evidence of malignant cells in ascitic fluid even after examination twice. Ultrasonography of the abdomen and pelvis was also unremarkable except for gross ascites. Besides this, the patient had undergone abdominal surgery for LSCS four months back after which abdominal distension started. This could have been the source of the spread of tubercular bacilli to the peritoneum from a hidden source of TB like the female reproductive tract, which may have occurred due to the low immune status of the patient and recent surgery. The diagnosis of tuberculous peritonitis was made based on history and raised ADA levels in ascitic fluid, and treatment with anti-tubercular therapy was successful. So, peritoneal TB should always be considered as a differential diagnosis while encountering a case of a female patient with ascites and elevated serum CA-125, especially in TB-endemic regions.
